# Adjuvanted Recombinant Zoster Vaccine is Effective Against Herpes Zoster Ophthalmicus, and is Associated With Lower Risk of Acute Myocardial Infarction and Stroke in Adults Aged ≥50 Years

**DOI:** 10.1093/cid/ciaf440

**Published:** 2025-08-09

**Authors:** Emily Rayens, Lina S Sy, Lei Qian, Jun Wu, Bradley K Ackerson, Yi Luo, Chengyi Zheng, Yanjun Cheng, Leticia I Vega Daily, Jeannie Song, Harpreet S Takhar, Jennifer H Ku, Rachel A Cohen, Huifeng Yun, Driss Oraichi, Harry Seifert, Hung-Fu Tseng

**Affiliations:** Department of Research & Evaluation, Kaiser Permanente Southern California, Pasadena, California, USA; Department of Research & Evaluation, Kaiser Permanente Southern California, Pasadena, California, USA; Department of Research & Evaluation, Kaiser Permanente Southern California, Pasadena, California, USA; Department of Research & Evaluation, Kaiser Permanente Southern California, Pasadena, California, USA; Department of Research & Evaluation, Kaiser Permanente Southern California, Pasadena, California, USA; Department of Research & Evaluation, Kaiser Permanente Southern California, Pasadena, California, USA; Department of Research & Evaluation, Kaiser Permanente Southern California, Pasadena, California, USA; Department of Research & Evaluation, Kaiser Permanente Southern California, Pasadena, California, USA; Department of Research & Evaluation, Kaiser Permanente Southern California, Pasadena, California, USA; Department of Research & Evaluation, Kaiser Permanente Southern California, Pasadena, California, USA; Department of Research & Evaluation, Kaiser Permanente Southern California, Pasadena, California, USA; Center for Integrated Health Care Research, Kaiser Permanente Hawaii, Honolulu, Hawaii, USA; GSK, Rockville, Maryland, USA; GSK, Rockville, Maryland, USA; GSK, Rockville, Maryland, USA; GSK, Rockville, Maryland, USA; Department of Research & Evaluation, Kaiser Permanente Southern California, Pasadena, California, USA; Kaiser Permanente Bernard J. Tyson School of Medicine, Pasadena, California, USA

**Keywords:** herpes zoster, recombinant zoster vaccine, herpes zoster ophthalmicus, hospitalized stroke, hospitalized acute myocardial infarction

## Abstract

**Background:**

Herpes zoster (HZ) and HZ ophthalmicus (HZO) are associated with an increased risk of cardiovascular complications, such as acute myocardial infarction (AMI) and stroke. We evaluated the association between recombinant zoster vaccine (RZV) and risk of HZO, hospitalized AMI, and hospitalized stroke in adults ≥50 years of age (YoA) at Kaiser Permanente Southern California.

**Methods:**

We conducted a matched cohort analysis of adults ≥50 YoA who received 2 doses of RZV 4 weeks–6 months apart during 01 April 2018–31 December 2020 and were matched 1:4 to RZV-unvaccinated individuals on age, sex, race/ethnicity, and index date (date of second dose among vaccinated; unvaccinated match assigned same date). Follow-up started at index date and ended at receipt of zoster vaccine, termination of membership, death, or 31 December 2022, whichever came first. HZO was identified by natural language processing of clinical notes; hospitalized AMI and stroke were identified by ICD-10 codes. Stratified Cox proportional hazards regression was used to estimate adjusted hazard ratios (aHR) and 95% confidence intervals (CI).

**Results:**

Among 102 766 2-dose RZV-vaccinated and 411 064 unvaccinated adults, 59.0% were female, 57.1% non-Hispanic White, and median age was 68 years (range: 50–108). The aHRs (95% CI) of HZO, hospitalized AMI, and hospitalized stroke comparing RZV-vaccinated and unvaccinated individuals were 0.271 (95% CI: 0.222, 0.330), 0.720 (0.588–0.881), and 0.575 (0.533–0.619), respectively. The adjusted RZV effectiveness against HZO was 72.9% (67.0%–77.8%).

**Conclusions:**

Among adults ≥50 YoA, 2 RZV doses were associated with a lower risk of HZO, AMI, and stroke.

## Teaser

In adults ≥50 years, adjusted effectiveness of 2 recombinant zoster vaccine doses against herpes zoster ophthalmicus was 72.9% (67.0%–77.8%). Recombinant zoster vaccine was associated with a reduced risk of hospitalized acute myocardial infarction and stroke (adjusted hazard ratios: 0.720–0.575).

Herpes zoster (HZ), or shingles, is an often painful and debilitating vesicular rash caused by reactivation of varicella zoster virus persisting latently in dorsal root ganglia [1, 2]. Some cases of HZ manifest as HZ ophthalmicus (HZO) [3], which can lead to ocular complications and loss of vision, particularly without antiviral treatment. The risk of severe HZ and associated complications increases with age, with incidence rates (IRs) sharply increasing after 50 years of age (YoA). HZ and HZO are associated with an increased risk of cardiovascular complications, such as acute myocardial infarction (AMI) and stroke [4–7]. Retrospective studies have reported a consistently higher risk of AMI in adults with HZ compared to those without HZ [4, 5]. Other retrospective analyses provided additional evidence that HZO is associated with an increased risk of AMI and stroke, and that this risk is highest in the first month [8] or the first year [4, 9] after the HZ or HZO episode, which is corroborated by reviews and meta-analyses [6, 7, 10–12].

Accordingly, and with some exceptions [13], observational studies have shown that immunization against HZ was associated with lower risks of AMI and/or stroke [14–18]. Of note, most data were obtained from adults immunized with the live-attenuated zoster vaccine (ZVL; *Zostavax*, Merck). In 2017, recombinant zoster vaccine (RZV; *Shingrix*, GSK) was approved by the US Food and Drug Administration for prevention of HZ in all adults ≥50 YoA and adults ≥18 YoA who are or will be at increased risk of the disease due to immunodeficiency/immunosuppression caused by known disease or therapy [19]. To date, 3 studies have reported a significantly reduced risk of AMI and/or stroke following immunization with RZV [5, 16, 20]. Two other studies did not report significant risk reduction [21, 22].

In phase III randomized, controlled trials, RZV showed an acceptable safety profile and high efficacy against HZ: 97.2% (95% confidence interval [CI]: 93.7%–99.0%) in ≥50 YoA and 89.8% (84.2%–93.7%) in ≥70 YoA individuals [23, 24]. In real-world observational studies, the vaccine effectiveness (VE) of RZV against HZ and postherpetic neuralgia (PHN) ranged between 70.1% (68.6%–71.5%) and 87.6% (78.9%–92.7%) in adults ≥50 YoA, depending on the age group analyzed [25–29]. Data on effectiveness of RZV against HZO are limited to 3 studies, with a reported 2-dose VE ranging between 66.8% for individuals ≥65 YoA, including immunocompromised persons, and 93.3% for individuals ≥50 YoA, excluding immunocompromised persons [26, 27, 30]. The present study aimed to evaluate the association between RZV and reduced risk of HZO, AMI, and stroke, and to evaluate the VE of RZV against HZO, in adults ≥50 YoA at Kaiser Permanente Southern California (KPSC).

## METHODS

### Study Setting

KPSC is a large, integrated healthcare system that includes >4.6 million members with diverse sociodemographic characteristics, reflecting the Southern California population. Membership retention rates are stable and 93% of members remain with KPSC for longer than a year [31]. Comprehensive membership and healthcare information, including demographics, vaccinations, diagnoses, and pharmacy records of KPSC members are stored in electronic health records (EHR). KPSC is a prepaid healthcare system and recommended vaccines such as RZV are provided to KPSC members at no charge. Vaccinations received outside of KPSC with appropriate documentation are also incorporated into the KPSC EHR.

This study was approved by the KPSC Institutional Review Board, with a waiver for written informed consent as the proposed use of EHR data involved minimal risk.

### Study Design and Population

We conducted an observational matched cohort study [32]. Eligible KPSC members were ≥50 YoA at index date (defined below) with continuous KPSC membership (allowing for a 31-day gap) in the year prior to index date. The RZV-vaccinated cohort consisted of individuals who received 2 doses of RZV 4 weeks–6 months apart between 1 April 2018 and 31 December 2020. Each vaccinated individual was matched with up to 4 RZV-unvaccinated individuals (unvaccinated cohort) by age, sex, and race/ethnicity. For vaccinated individuals, the index date was defined as the date of vaccination with the second dose of RZV, and unvaccinated individuals were assigned the same index date as their vaccinated matches. Individuals were excluded if they received the second dose of RZV <4 weeks after the first or if they were diagnosed with HZ in the 6 months prior to or within 30 days after the index date. Individuals diagnosed with HZ in the 30 days after the index date were excluded to avoid misclassification of subclinical HZ and to allow sufficient time for the second RZV dose to take effect. Individuals with prior ZVL immunization were not excluded, but this characteristic was considered as a covariate in the analysis [32].

Individuals were followed from the index date until death, receipt of an RZV or ZVL dose during follow-up (for unvaccinated individuals), receipt of an additional RZV or ZVL dose during follow-up (for vaccinated individuals), termination of KPSC membership (allowing for a 31-day gap in membership), occurrence of the outcome of interest, or 31 December 2022 (for the present interim analysis), whichever came first. The total planned follow-up time for this study is 10 years, until 31 December 2030, and the results presented in this manuscript are part of an interim analysis.

### Objectives

The primary and several secondary objectives, pertaining to long-term RZV VE against HZ and PHN, have been reported in a related manuscript [32]. The secondary objectives reported here were to estimate the VE of 2 RZV doses in preventing HZO, as well as to evaluate the association of 2 doses of RZV with reduced risk of HZO, hospitalized AMI, and hospitalized stroke in adults ≥50 YoA.

### Exposure and Outcomes

RZV vaccine doses were identified using CVX (vaccine administered) code 187: zoster vaccine recombinant. HZO during the follow-up period was identified using natural language processing (NLP) of EHR free-text notes, as described previously [33]. Hospitalized AMI and stroke were identified by International Classification of Diseases 10th Revision (ICD-10) codes from inpatient encounters, or Emergency Department encounters if the patient died the same or next day (Supplementary material, Supplementary Table 1). Individuals with AMI and stroke diagnosis codes within 183 days prior to the index date were excluded.

### Statistical Methods and Analysis

The IRs of outcomes were calculated by dividing the number of cases by the total number of person-years of follow-up (PY). Adjusted hazard ratios (aHRs) and 95% CIs comparing IRs in 2-dose RZV and unvaccinated individuals were estimated using Cox proportional hazards regression models, adjusting for potential confounders determined by scientific relevance and absolute standardized difference >0.1 [32]. In a post hoc analysis, results were stratified by history of ZVL vaccination. Adjusted VE (%) against HZO was calculated as (1 − aHR) × 100 if aHR ≤1 and ([1/aHR] − 1) × 100 if aHR >1. Full details of the statistical analysis are presented in the accompanying publication [32 ].

## RESULTS

### Study Participants

In total, 102 766 and 411 064 individuals were included in the 2-dose (4 weeks–6 months) RZV and matched unvaccinated cohort, respectively. The median age of included individuals was 68 (range: 50–108) years; 59.0% were female and 57.1% were non-Hispanic White. The mean follow-up time was 2.5 years. The complete baseline characteristics of the study cohorts are detailed in the related manuscript [32].

### Outcomes of Interest

Among individuals who received 2 doses of RZV, there were 121 HZO cases with an IR of 0.4 (95% CI: 0.3–0.5) per 1000 PY compared to 1334 HZO cases with an IR of 1.4 (1.3–1.5) per 1000 PY in the unvaccinated group (Table 1). The aHR was 0.271 (0.222–0.330) and the overall adjusted RZV VE against HZO was 72.9% (67.0%–77.8%) (Table 1). In the vaccinated group, there were 153 cases of hospitalized AMI (IR: 0.5 [0.4–0.6] per 1000 PY) and 989 cases of hospitalized stroke (IR: 3.3 [3.1–3.5] per 1000 PY), compared to 619 cases of hospitalized AMI (IR: 0.6 [0.6–0.7] per 1000 PY) and 5306 cases of hospitalized stroke (IR: 5.5 [5.4–5.7] per 1000 PY) in the unvaccinated group (Table 1). The overall aHRs of hospitalized AMI and stroke were 0.720 (0.588–0.881) and 0.575 (0.533–0.619) (Table 1).

**Table 1.** Incidence Rate, Hazard Ratio, and Effectiveness of 2 Doses of RZV in Preventing HZO, Hospitalized AMI, and Hospitalized Stroke

Outcomes	Vaccinated				Unvaccinated				HR (95% CI)		VE, % (95% CI)^a^	
	N	n	PY	IR/1000 PY (95% CI)	N	n	PY	IR/1000 PY (95% CI)	Unadjusted	Adjusted^b^	Unadjusted	Adjusted^b^
HZO	102 766	121	299 914.4	0.4 (0.3–0.5)	411 064	1334	959 934.5	1.4 (1.3–1.5)	0.285 (0.235–0.345)	0.271 (0.222–0.330)	71.5 (65.5–76.5)	72.9 (67.0–77.8)
Hospitalized AMI^c^	102 526	153	300 308.9	0.5 (0.4–0.6)	409 522	619	967 051.1	0.6 (0.6–0.7)	0.737 (0.614–0.886)	0.720 (0.588–0.881)	N/A	N/A
Hospitalized stroke^c^	102 303	989	298 785.6	3.3 (3.1–3.5)	408 337	5306	960 122.1	5.5 (5.4–5.7)	0.577 (0.538–0.619)	0.575 (0.533–0.619)	N/A	N/A

The 2 RZV doses were administered 4 wks–6 m apart.

AMI, acute myocardial infarction; CI, confidence interval; HR, hazard ratio; HZ, herpes zoster; HZO, herpes zoster ophthalmicus; IR, incidence rate; N, number of assessed individuals in each cohort; n, number of individuals with a given outcome; N/A, not assessed; PY, person-years; RZV, adjuvanted recombinant zoster vaccine (*Shingrix*, GSK); VE, vaccine effectiveness; ZVL, live-attenuated zoster vaccine (*Zostavax*, Merck).

^a^VE (%) = (1 − HR) × 100 if HR ≤1, and VE (%) = ([1/HR] − 1) × 100 if HR >1.

^b^Adjusted for covariates: history of HZ, number of outpatient/virtual visits, history of ZVL, and length of continuous membership at baseline; time-varying immunocompromised status and heart disease during follow-up period.

^c^Individuals with AMI or stroke in the 6 m prior to the index date were excluded from the cohorts for their respective analyses; VE against hospitalized AMI and stroke was not calculated.

In a post hoc analysis, there was no significant difference in aHR and VE of two doses of RZV in preventing HZO or aHR for hospitalized AMI and hospitalized stroke when stratified by ZVL history (Supplementary Table 2). A plain language summary of the results for nonexpert audiences is available in Supplementary Figure 1.

## DISCUSSION

This study found that 2 doses of RZV were effective in preventing HZO in adults ≥50 YoA and were associated with significantly reduced risk of hospitalized AMI and stroke compared to unvaccinated individuals. These data suggest that health benefits of RZV immunization may extend beyond the well-described prevention of HZ and PHN. This is one of the first real-world studies contributing evidence on RZV immunization and the reduced risk of HZO, hospitalized AMI, and hospitalized stroke.

The IR of HZO in RZV-vaccinated versus unvaccinated individuals (IR: 0.4 vs 1.4 per 1000 PY) observed in this study was comparable to data previously reported for 2-dose RZV-vaccinated versus unvaccinated individuals (0.1 vs 0.7 and 0.3 vs 0.8 per 1000 PY) [26, 27, 30]. The observed VE of 2 doses of RZV against HZO was also comparable at 72.9%, with previous reports ranging between 66.8% and 93.3% [26, 27, 30]. Lower VE was reported in designs that included immunocompromised patient populations, as included in our study, and in those that focused on older patient populations. At this time, most existing data evaluating HZ vaccination related to AMI and stroke were obtained in studies on ZVL immunization [13 , 14 , 16]. However, an analysis of patients who received care at a Veteran Affairs facility found a decreased risk of stroke in patients who received either RZV or ZVL, with a stronger effect observed with RZV (odds ratio of RZV 0.57 [95% CI: 0.46–0.72; *P* < .001]; ZVL 0.77 [95% CI: 0.65–0.91; *P* = .002]) [16]. Here, we report a 28.0% and 42.5% reduction in risk of all-cause hospitalized AMI and stroke associated with 2 doses of RZV.

The study had several notable strengths. First, KPSC's comprehensive EHR allows for accurate capture of RZV vaccinations, covariates, and outcomes. Second, the use of NLP to identify HZO captures significantly more cases than those identified by diagnosis codes alone, with high sensitivity and specificity, thereby improving confidence in this outcome [33]. Third, the vaccinated study population was large and increased power for statistical analysis of HZO, hospitalized AMI, and hospitalized stroke outcomes. Fourth, KPSC members were diverse in age, race/ethnicity, and health status, representing the general population recommended for vaccination. Fifth, the vaccine was available to KPSC members at no cost, increasing the likelihood of receipt within the KPSC system and minimizing exposure misclassification.

This study also had some limitations. First, although the analyses were adjusted for demographic and clinical covariates, residual confounding from covariates not included in the analyses might still exist. We included prior healthcare utilization as a covariate because differential healthcare-seeking behaviors may cause misclassification of HZO. Second, this analysis did not allow for calculation of RZV effectiveness against HZ-related stroke or AMI; currently, we can only report associated reduced risk of all-cause stroke and AMI. At this time, it is possible that the observed reduction in risk may not solely be explained by the prevention of HZ by RZV vaccination. As the cohort follow-up continues, a sufficient number of hospitalized AMI or stroke cases up to 3 months following HZ infection, which were not collected for this publication, may accrue, allowing estimation of aHR and VE to assess RZV and reduced risk of hospitalized AMI or stroke after HZ diagnosis. Third, it is possible that use of diagnosis code-based identification of AMI and stroke resulted in outcome misclassification. However, the positive predictive value of ICD-10 codes to identify these outcomes is relatively high [34–36].

This study provides real-world evidence that a 2-dose RZV vaccination schedule is effective in preventing HZO (VE [95% CI]: 72.9% [67.0%–77.8%]) and is associated with a lower risk of hospitalized AMI and hospitalized stroke (aHR [95% CI]: 0.720 [0.588–0.881] and 0.575 [0.533–0.619], respectively). These findings demonstrate that there may be additional benefits of RZV vaccination for reducing the risk of HZ-associated outcomes beyond HZ and PHN.

## Supplementary Material

ciaf440_Supplementary_Data

## Supplementary Data

Supplementary materials are available at *Clinical Infectious Diseases* online. Consisting of data provided by the authors to benefit the reader, the posted materials are not copyedited and are the sole responsibility of the authors, so questions or comments should be addressed to the corresponding author.
