# Supplementary material for: Adjuvanted Recombinant Zoster Vaccine is Effective Against Herpes Zoster Ophthalmicus, and is Associated With Lower Risk of Acute Myocardial Infarction and Stroke in Adults Aged ≥50 Years
Source: Clin Infect Dis. 2025 Aug 9;81(5):e441–5. doi: 10.1093/cid/ciaf440 (PMC12728275; doi:10.1093/cid/ciaf440)

**Title:** Adjuvanted recombinant zoster vaccine is effective against herpes zoster ophthalmicus, and is associated with lower risk of acute myocardial infarction and stroke in adults aged ≥50 years

Emily Rayens, Lina S. Sy, Lei Qian, Jun Wu, Bradley K. Ackerson, Yi Luo, Chengyi Zheng, Yanjun Cheng, Leticia I. Vega Daily, Jeannie Song, Harpreet S. Takhar, Jennifer H. Ku, Rachel A. Cohen, Huifeng Yun, Driss Oraichi, Harry Seifert, Hung-Fu Tseng

**Supplementary material**

Table of Contents

[Outcome definition details 2](#_Toc203725536)

[Herpes zoster ophthalmicus (HZO) 2](#_Toc203725537)

[Hospitalized acute myocardial infarction (AMI) 2](#_Toc203725538)

[Hospitalized stroke 2](#_Toc203725539)

[Covariates 2](#_Toc203725540)

[Supplementary Table 1. Outcome definitions by the International Classification of Diseases 10th Revision (ICD-10) codes. 4](#_Toc203725541)

[Supplementary Table 2. Incidence rate, hazard ratio, and VE of 2 doses (4 weeks to 6 months) of RZV in preventing outcomes stratified by history of ZVL. 7](#_Toc203725542)

[Supplementary Figure 1. Plain Language Summary 8](#_Toc203725543)

# Outcome definition details

## Herpes zoster ophthalmicus (HZO)

HZO during the follow-up period was identified among incident herpes zoster (HZ) events using a natural language processing (NLP) algorithm. The NLP algorithm was applied on clinical notes from 7 days before to 30 days after the incident HZ event date.

## Hospitalized acute myocardial infarction (AMI)

Hospitalized AMI was defined as at least one AMI diagnosis code in the primary diagnosis position from the hospital setting or, if the patient died the same or the next day, from the Emergency Department (ED) setting during follow-up. Individuals with an AMI record in the 183 days prior to the index date were excluded from the cohort for AMI analyses. The diagnosis date of the first eligible AMI diagnosis after the index date was defined as the AMI event date.

## Hospitalized stroke

Hospitalized stroke was defined as at least one hemorrhagic stroke diagnosis code in the primary diagnosis position from the hospital setting or, if the patient died the same or the next day, from the ED setting; or as at least one ischemic stroke diagnosis code in any position from the hospital setting or, if the patient died the same or the next day, from the ED setting during follow-up. Stroke events with a traumatic brain injury code in the primary diagnosis position from the hospital or ED setting on the same date as the stroke diagnosis were excluded. Individuals with stroke in the 183 days prior to the index date were excluded from the cohort for stroke analyses.

The diagnosis date of the first eligible stroke diagnosis after the index date was defined as the stroke event date.

# Covariates

Variables that were identified from electronic health records and considered in analyses when appropriate as covariates or stratification variables, included:

• Demographic variables: age at index date, sex, race/ethnicity

• Healthcare utilization (number of outpatient/emergency/inpatient encounters) in year prior to index date

• Comorbidities (e.g., kidney disease, heart disease, lung disease, liver disease, diabetes) in year prior to index date. Comorbidity status during follow-up was updated every 365 days after index date

• Immunocompromised status assessed at index date and during follow-up (time-varying)

• History of zoster vaccine live (ZVL) prior to index date

• History of HZ prior to index date

• Concomitant vaccinations at time of exposure (e.g., receipt of an influenza, pneumococcal, or tetanus-diphtheria-acellular pertussis vaccine)

• Length of continuous membership prior to index date

# Supplementary Table 1. Outcome definitions by the International Classification of Diseases 10th Revision (ICD-10) codes.

| **Condition** | **ICD-10 codes** | **Code description** |
| --- | --- | --- |
| **Acute myocardial infarction** | I21.01 | STEMI involving left main coronary artery |
|  | I21.02 | STEMI involving left anterior descending coronary artery |
|  | I21.09 | STEMI involving other coronary artery of anterior wall |
|  | I21.11 | STEMI involving right coronary artery |
|  | I21.19 | STEMI involving other coronary artery of inferior wall |
|  | I21.21 | STEMI involving left circumflex coronary artery |
|  | I21.29 | STEMI involving other sites |
|  | I21.3 | STEMI of unspecified site |
|  | I21.4 | Non-STEMI |
|  | I21.9 | Acute myocardial infarction, unspecified |
|  | I21.A1 | Myocardial infarction type 2 |
|  | I21.A9 | Other myocardial infarction type |
|  | I22.0 | Subsequent STEMI of anterior wall |
|  | I22.1 | Subsequent STEMI of inferior wall |
|  | I22.2 | Subsequent non-STEMI |
|  | I22.8 | Subsequent STEMI of other sites |
|  | I22.9 | Subsequent STEMI of unspecified site |
| **Hemorrhagic stroke** | I60.00 | Nontraumatic subarachnoid hemorrhage from unspecified carotid siphon and bifurcation |
|  | I60.01 | Nontraumatic subarachnoid hemorrhage from right carotid siphon and bifurcation |
|  | I60.02 | Nontraumatic subarachnoid hemorrhage from left carotid siphon and bifurcation |
|  | I60.10 | Nontraumatic subarachnoid hemorrhage from unspecified middle cerebral artery |
|  | I60.11 | Nontraumatic subarachnoid hemorrhage from right middle cerebral artery |
|  | I60.12 | Nontraumatic subarachnoid hemorrhage from left middle cerebral artery |
|  | I60.2 | Nontraumatic subarachnoid hemorrhage from anterior communicating artery |
|  | I60.30 | Nontraumatic subarachnoid hemorrhage from unspecified posterior communicating artery |
|  | I60.31 | Nontraumatic subarachnoid hemorrhage from right posterior communicating artery |
|  | I60.32 | Nontraumatic subarachnoid hemorrhage from left posterior communicating artery |
|  | I60.4 | Nontraumatic subarachnoid hemorrhage from basilar artery |
|  | I60.50 | Nontraumatic subarachnoid hemorrhage from unspecified vertebral artery |
|  | I60.51 | Nontraumatic subarachnoid hemorrhage from right vertebral artery |
|  | I60.52 | Nontraumatic subarachnoid hemorrhage from left vertebral artery |
|  | I60.6 | Nontraumatic subarachnoid hemorrhage from other intracranial arteries |
|  | I60.7 | Nontraumatic subarachnoid hemorrhage from unspecified intracranial artery |
|  | I60.8 | Other nontraumatic subarachnoid hemorrhage |
|  | I60.9 | Nontraumatic subarachnoid hemorrhage, unspecified |
|  | I61.0 | Nontraumatic intracerebral hemorrhage in hemisphere, subcortical |
|  | I61.1 | Nontraumatic intracerebral hemorrhage in hemisphere, cortical |
|  | I61.2 | Nontraumatic intracerebral hemorrhage in hemisphere, unspecified |
|  | I61.3 | Nontraumatic intracerebral hemorrhage in brain stem |
|  | I61.4 | Nontraumatic intracerebral hemorrhage in cerebellum |
|  | I61.5 | Nontraumatic intracerebral hemorrhage, intraventricular |
|  | I61.6 | Nontraumatic intracerebral hemorrhage, multiple localized |
|  | I61.8 | Other nontraumatic intracerebral hemorrhage |
|  | I61.9 | Nontraumatic intracerebral hemorrhage, unspecified |
| **Ischemic stroke** | I63.00 | Cerebral infarction due to thrombosis of unspecified precerebral artery |
|  | I63.011 | Cerebral infarction due to thrombosis of right vertebral artery |
|  | I63.012 | Cerebral infarction due to thrombosis of left vertebral artery |
|  | I63.013 | Cerebral infarction due to thrombosis of bilateral vertebral arteries |
|  | I63.019 | Cerebral infarction due to thrombosis of unspecified vertebral artery |
|  | I63.02 | Cerebral infarction due to thrombosis of basilar artery |
|  | I63.031 | Cerebral infarction due to thrombosis of right carotid artery |
|  | I63.032 | Cerebral infarction due to thrombosis of left carotid artery |
|  | I63.033 | Cerebral infarction due to thrombosis of bilateral carotid arteries |
|  | I63.039 | Cerebral infarction due to thrombosis of unspecified carotid artery |
|  | I63.09 | Cerebral infarction due to thrombosis of other precerebral artery |
|  | I63.10 | Cerebral infarction due to embolism of unspecified precerebral artery |
|  | I63.111 | Cerebral infarction due to embolism of right vertebral artery |
|  | I63.112 | Cerebral infarction due to embolism of left vertebral artery |
|  | I63.113 | Cerebral infarction due to embolism of bilateral vertebral arteries |
|  | I63.119 | Cerebral infarction due to embolism of unspecified vertebral artery |
|  | I63.12 | Cerebral infarction due to embolism of basilar artery |
|  | I63.131 | Cerebral infarction due to embolism of right carotid artery |
|  | I63.132 | Cerebral infarction due to embolism of left carotid artery |
|  | I63.133 | Cerebral infarction due to embolism of bilateral carotid arteries |
|  | I63.139 | Cerebral infarction due to embolism of unspecified carotid artery |
|  | I63.19 | Cerebral infarction due to embolism of other precerebral artery |
|  | I63.20 | Cerebral infarction due to unspecified occlusion or stenosis of unspecified precerebral arteries |
|  | I63.211 | Cerebral infarction due to unspecified occlusion or stenosis of right vertebral artery |
|  | I63.212 | Cerebral infarction due to unspecified occlusion or stenosis of left vertebral artery |
|  | I63.213 | Cerebral infarction due to unspecified occlusion or stenosis of bilateral vertebral arteries |
|  | I63.219 | Cerebral infarction due to unspecified occlusion or stenosis of unspecified vertebral artery |
|  | I63.22 | Cerebral infarction due to unspecified occlusion or stenosis of basilar artery |
|  | I63.231 | Cerebral infarction due to unspecified occlusion or stenosis of right carotid arteries |
|  | I63.232 | Cerebral infarction due to unspecified occlusion or stenosis of left carotid arteries |
|  | I63.233 | Cerebral infarction due to unspecified occlusion or stenosis of bilateral carotid arteries |
|  | I63.239 | Cerebral infarction due to unspecified occlusion or stenosis of unspecified carotid artery |
|  | I63.29 | Cerebral infarction due to unspecified occlusion or stenosis of other precerebral arteries |
|  | I63.30 | Cerebral infarction due to thrombosis of unspecified cerebral artery |
|  | I63.311 | Cerebral infarction due to thrombosis of right middle cerebral artery |
|  | I63.312 | Cerebral infarction due to thrombosis of left middle cerebral artery |
|  | I63.313 | Cerebral infarction due to thrombosis of bilateral middle cerebral arteries |
|  | I63.319 | Cerebral infarction due to thrombosis of unspecified middle cerebral artery |
|  | I63.321 | Cerebral infarction due to thrombosis of right anterior cerebral artery |
|  | I63.322 | Cerebral infarction due to thrombosis of left anterior cerebral artery |
|  | I63.323 | Cerebral infarction due to thrombosis of bilateral anterior cerebral arteries |
|  | I63.329 | Cerebral infarction due to thrombosis of unspecified anterior cerebral artery |
|  | I63.331 | Cerebral infarction due to thrombosis of right posterior cerebral artery |
|  | I63.332 | Cerebral infarction due to thrombosis of left posterior cerebral artery |
|  | I63.333 | Cerebral infarction due to thrombosis of bilateral posterior cerebral arteries |
|  | I63.339 | Cerebral infarction due to thrombosis of unspecified posterior cerebral artery |
|  | I63.341 | Cerebral infarction due to thrombosis of right cerebellar artery |
|  | I63.342 | Cerebral infarction due to thrombosis of left cerebellar artery |
|  | I63.343 | Cerebral infarction due to thrombosis of bilateral cerebellar arteries |
|  | I63.349 | Cerebral infarction due to thrombosis of unspecified cerebellar artery |
|  | I63.39 | Cerebral infarction due to thrombosis of other cerebral artery |
|  | I63.40 | Cerebral infarction due to embolism of unspecified cerebral artery |
|  | I63.411 | Cerebral infarction due to embolism of right middle cerebral artery |
|  | I63.412 | Cerebral infarction due to embolism of left middle cerebral artery |
|  | I63.413 | Cerebral infarction due to embolism of bilateral middle cerebral arteries |
|  | I63.419 | Cerebral infarction due to embolism of unspecified middle cerebral artery |
|  | I63.421 | Cerebral infarction due to embolism of right anterior cerebral artery |
|  | I63.422 | Cerebral infarction due to embolism of left anterior cerebral artery |
|  | I63.423 | Cerebral infarction due to embolism of bilateral anterior cerebral arteries |
|  | I63.429 | Cerebral infarction due to embolism of unspecified anterior cerebral artery |
|  | I63.431 | Cerebral infarction due to embolism of right posterior cerebral artery |
|  | I63.432 | Cerebral infarction due to embolism of left posterior cerebral artery |
|  | I63.433 | Cerebral infarction due to embolism of bilateral posterior cerebral arteries |
|  | I63.439 | Cerebral infarction due to embolism of unspecified posterior cerebral artery |
|  | I63.441 | Cerebral infarction due to embolism of right cerebellar artery |
|  | I63.442 | Cerebral infarction due to embolism of left cerebellar artery |
|  | I63.443 | Cerebral infarction due to embolism of bilateral cerebellar arteries |
|  | I63.449 | Cerebral infarction due to embolism of unspecified cerebellar artery |
|  | I63.49 | Cerebral infarction due to embolism of other cerebral artery |
|  | I63.50 | Cerebral infarction due to unspecified occlusion or stenosis of unspecified cerebral artery |
|  | I63.511 | Cerebral infarction due to unspecified occlusion or stenosis of right middle cerebral artery |
|  | I63.512 | Cerebral infarction due to unspecified occlusion or stenosis of left middle cerebral artery |
|  | I63.513 | Cerebral infarction due to unspecified occlusion or stenosis of bilateral middle cerebral arteries |
|  | I63.519 | Cerebral infarction due to unspecified occlusion or stenosis of unspecified middle cerebral artery |
|  | I63.521 | Cerebral infarction due to unspecified occlusion or stenosis of right anterior cerebral artery |
|  | I63.522 | Cerebral infarction due to unspecified occlusion or stenosis of left anterior cerebral artery |
|  | I63.523 | Cerebral infarction due to unspecified occlusion or stenosis of bilateral anterior cerebral arteries |
|  | I63.529 | Cerebral infarction due to unspecified occlusion or stenosis of unspecified anterior cerebral artery |
|  | I63.531 | Cerebral infarction due to unspecified occlusion or stenosis of right posterior cerebral artery |
|  | I63.532 | Cerebral infarction due to unspecified occlusion or stenosis of left posterior cerebral artery |
|  | I63.533 | Cerebral infarction due to unspecified occlusion or stenosis of bilateral posterior cerebral arteries |
|  | I63.539 | Cerebral infarction due to unspecified occlusion or stenosis of unspecified posterior cerebral artery |
|  | I63.541 | Cerebral infarction due to unspecified occlusion or stenosis of right cerebellar artery |
|  | I63.542 | Cerebral infarction due to unspecified occlusion or stenosis of left cerebellar artery |
|  | I63.543 | Cerebral infarction due to unspecified occlusion or stenosis of bilateral cerebellar arteries |
|  | I63.549 | Cerebral infarction due to unspecified occlusion or stenosis of unspecified cerebellar artery |
|  | I63.59 | Cerebral infarction due to unspecified occlusion or stenosis of other cerebral artery |
|  | I63.6 | Cerebral infarction due to cerebral venous thrombosis, nonpyogenic |
|  | I63.8 | Other cerebral infarction |
|  | I63.81 | Other cerebral infarction due to occlusion or stenosis of small artery |
|  | I63.89 | Other cerebral infarction |
|  | I63.9 | Cerebral infarction, unspecified |
| STEMI, ST elevation myocardial infarction. | | |

# **Supplementary Table 2.** Incidence rate, hazard ratio, and VE of 2 doses (4 weeks to 6 months) of RZV in preventing outcomes stratified by history of ZVL.

|  | **Vaccinated** | | | |  | **Unvaccinated** | | | |  | **Hazard Ratio (95% CI)** | | | |  | **VE (95% CI)^a^** | |
| --- | --- | --- | --- | --- | --- | --- | --- | --- | --- | --- | --- | --- | --- | --- | --- | --- | --- |
| **Outcomes** | **N** | **Number of cases** | **Number of person years** | **Incidence per 1000 person-years  (95% CI)** |  | **N** | **Number of cases** | **Number of person years** | **Incidence per 1000 person-years (95% CI)** |  | | **Unadjusted** | | **Adjusted^b^** |  | **Unadjusted** | **Adjusted^b^** |
| HZO | 102766 | 121 | 299914.4 | 0.4  (0.3, 0.5) |  | 411064 | 1334 | 959934.5 | 1.4  (1.3, 1.5) |  | 0.285  (0.235, 0.345) | | 0.271 (0.222, 0.330) | |  | 71.5% (65.5%, 76.5%) | 72.9% (67.0%, 77.8%) |
| History of ZVL, No^c^ | 54738 | 62 | 153899.5 | 0.4  (0.3, 0.5) |  | 295519 | 979 | 706021.1 | 1.4  (1.3, 1.5) |  | 0.291  (0.225, 0.376) | | 0.278 (0.214, 0.360) | |  | 70.9% (62.4%, 77.5%) | 72.2% (64.0%, 78.6%) |
| History of ZVL, Yes^c^ | 48028 | 59 | 146014.9 | 0.4  (0.3, 0.5) |  | 115545 | 355 | 253913.4 | 1.4  (1.3, 1.6) |  | 0.289  (0.219, 0.380) | | 0.281 (0.213, 0.371) | |  | 71.1% (62.0%, 78.1%) | 71.9% (62.9%, 78.7%) |
| Hospitalized AMI | 102526 | 153 | 300308.9 | 0.5  (0.4, 0.6) |  | 409522 | 619 | 967051.1 | 0.6  (0.6, 0.7) |  | 0.737  (0.614, 0.886) | | 0.720 (0.588, 0.881) | |  | N/A | N/A |
| History of ZVL, No^c^ | 54626 | 69 | 154128.7 | 0.4  (0.4, 0.6) |  | 294446 | 457 | 711510.0 | 0.6  (0.6, 0.7) |  | 0.675  (0.524, 0.870) | | 0.673 (0.521, 0.871) | |  | N/A | N/A |
| History of ZVL, Yes^c^ | 47900 | 84 | 146180.2 | 0.6  (0.5, 0.7) |  | 115076 | 162 | 255541.1 | 0.6  (0.5, 0.7) |  | 0.868  (0.666, 1.131) | | 0.797 (0.609, 1.041) | |  | N/A | N/A |
| Hospitalized stroke | 102303 | 989 | 298785.6 | 3.3  (3.1, 3.5) |  | 408337 | 5306 | 960122.1 | 5.5  (5.4, 5.7) |  | 0.577  (0.538, 0.619) | | 0.575 (0.533, 0.619) | |  | N/A | N/A |
| History of ZVL, No^c^ | 54519 | 390 | 153515.0 | 2.5  (2.3, 2.8) |  | 293733 | 3608 | 706980.5 | 5.1  (4.9, 5.3) |  | 0.491  (0.443, 0.546) | | 0.546 (0.491, 0.607) | |  | N/A | N/A |
| History of ZVL, Yes^c^ | 47784 | 599 | 145270.6 | 4.1  (3.8, 4.5) |  | 114604 | 1698 | 253141.6 | 6.7  (6.4, 7.0) |  | 0.598  (0.544, 0.656) | | 0.582 (0.529, 0.640) | |  | N/A | N/A |
| ^a^ VE (%) = (1 – HR) × 100 if HR ≤1, and VE (%) = ([1/HR] – 1) × 100 if HR >1. | | | | | | | | | | | | | | | | | |
| ^b^ Adjusted for covariates: history of HZ, number of outpatient/virtual visits, history of ZVL, and length of continuous membership at baseline; time-varying IC status and heart disease during follow-up period. | | | | | | | | | | | | | | | | | |
| ^c^ Adjusted for covariates: age group, sex, race/ethnicity, year and month of index date, history of HZ, number of outpatient/virtual visits, and length of continuous membership at baseline; time-varying IC status and heart disease during follow-up period.  AMI, acute myocardial infarction; CI, confidence interval; HZO, herpes zoster ophthalmicus; n, number; N/A, not applicable; RZV, recombinant zoster vaccine; ZVL, zoster vaccine live | | | | | | | | | | | | | | | | | |

# Supplementary Figure 1. Plain Language Summary


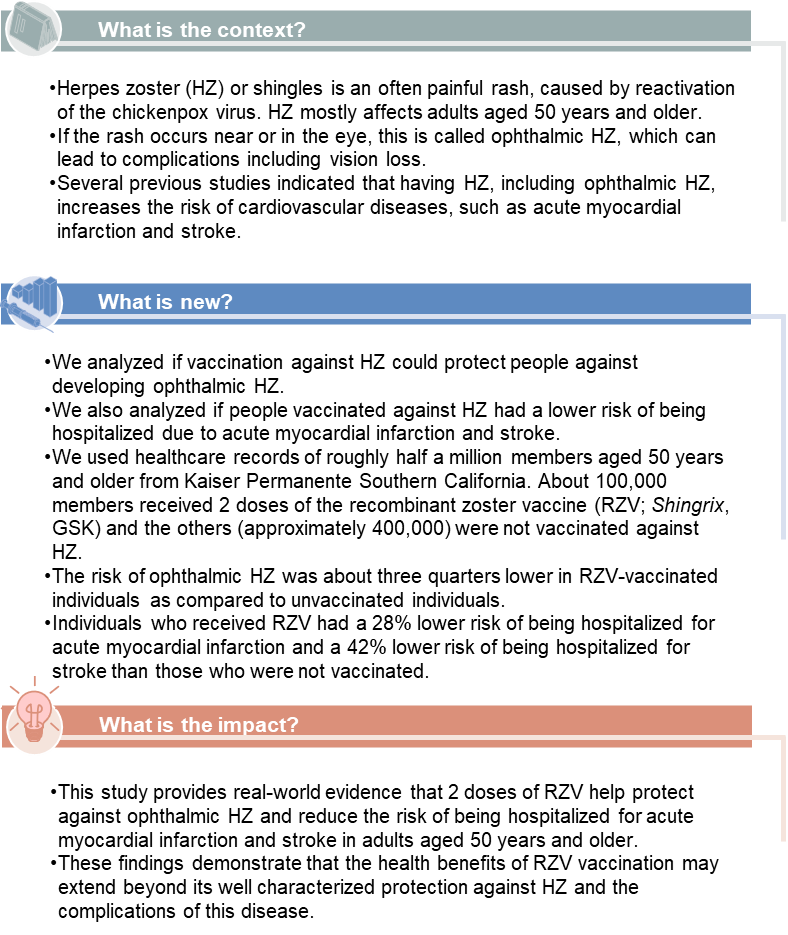

Supplement: ciaf440_Supplementary_Data [file ciaf440_supplementary_data.docx]
